# *Metarhizium anisopliae* infection reduces *Trypanosoma congolense* reproduction in *Glossina fuscipes fuscipes* and its ability to acquire or transmit the parasite

**DOI:** 10.1186/s12866-018-1277-6

**Published:** 2018-11-23

**Authors:** Lawrence G Wamiti, Fathiya M Khamis, Adly M M Abd-alla, Fidelis L O Ombura, Komivi S Akutse, Sevgan Subramanian, Samuel O Odiwuor, Shem J Ochieng, Sunday Ekesi, Nguya K Maniania

**Affiliations:** 1 International Centre of Insect Physiology and Ecology (icipe), P.O. Box 30772-00100, Nairobi, Kenya; 2 Mount Kenya University, P.O. Box 324-01000, Thika, Kenya; 3 Insect Pest Control Laboratory, Joint FAO/IAEA Division of Nuclear Techniques in Food and Agriculture, International Atomic Energy Agency, Wagramerstraße 5, A-1400 Vienna, Austria; 4 Medical Physiology Department, Kenyatta University, P.O. Box 43844-00100, Nairobi, Kenya

**Keywords:** Entomopathogenic fungus, Tsetse, Trypanosome, Parasite, Infection, Vector competence

## Abstract

**Background:**

Tsetse fly-borne trypanosomiasis remains a significant problem in Africa despite years of interventions and research. The need for new strategies to control and possibly eliminate trypanosomiasis cannot be over-emphasized. Entomopathogenic fungi (EPF) infect their hosts through the cuticle and proliferate within the body of the host causing death in about 3–14 days depending on the concentration. During the infection process, EPF can reduce blood feeding abilities in hematophagous arthropods such as mosquitoes, tsetse flies and ticks, which may subsequently impact the development and transmission of parasites. Here, we report on the effects of infection of tsetse fly (*Glossina fuscipes fuscipes*) by the EPF, *Metarhizium anisopliae* ICIPE 30 wild-type strain (WT) and green fluorescent protein-transformed strain (GZP-1) on the ability of the flies to harbor and transmit the parasite, *Trypanosoma congolense.*

**Results:**

Teneral flies were fed *T. congolense*-infected blood for 2 h and then infected using velvet carpet fabric impregnated with conidia covered inside a cylindrical plastic tube for 12 h. Control flies were fed with *T. congolense*-infected blood but not exposed to the fungal treatment via the carpet fabric inside a cylindrical plastic tube. Insects were dissected at 2, 3, 5 and 7 days post-fungal exposure and the density of parasites quantified. Parasite load decreased from 8.7 × 10^7^ at day 2 to between 8.3 × 10^4^ and 1.3 × 10^5^ *T. congolense* ml^− 1^ at day 3 post-fungal exposure in fungus-treated (WT and GZP-1) fly groups. When *T. congolense-*infected flies were exposed to either fungal strain, they did not transmit the parasite to mice whereas control treatment flies remained capable of parasite transmission. Furthermore, *M. anisopliae-*inoculated flies which fed on *T. congolense-*infected mice were not able to acquire the parasites at 4 days post-fungal exposure while parasite acquisition was observed in the control treatment during the same period.

**Conclusions:**

Infection of the vector *G. f. fuscipes* by the entomopathogenic fungus *M. anisopliae* negatively affected the multiplication of the parasite *T. congolense* in the fly and reduced the vectorial capacity to acquire or transmit the parasite*.*

## Background

Tsetse flies are prime vectors of protozoan parasites of the genus *Trypanosoma*, which cause sleeping sickness in humans (Human African Trypanosome-HAT) and ‘nagana’ in cattle (Animal African Trypanosome-AAT) in 36 countries in sub-Saharan Africa [1]. Annually, trypanosomiasis in sub-Saharan Africa costs approximately US $4.5 billion directly, leaving affected communities vulnerable to food shortages, starvation, famine and disease [2]. Cattand et al. [3] reported that 70 million people live at risk of infection but it is difficult to estimate the accurate disease prevalence since there are about 300,000 new cases reported per year, with many more cases probably remaining undetected or unreported. Current treatment of trypanosomiasis involves chemotherapeutic drugs that are toxic, with increasing levels of drug resistance emerging within the parasite populations [4, 5].

Trypanosomiasis management strategies continue to rely on control of the vector. These strategies include traps or targets impregnated with chemical insecticides [6–8], mass trapping [9–11], ‘pour on’ formulation applied to cattle [12, 13], and sterile insect technique [10, 14]. However, the potential application of the entomopathogenic fungus *Metarhizium anisopliae* (Metschn.) Sorok. in suppressing tsetse fly populations was recently demonstrated in the field using autodissemination devices mounted on pyramidal traps [15]. During the infection process, the incubation of entomopathogenic fungi (EPF) varies according to the host and environmental factors, with host death typically occuring within 3–7 days post-fungal infection. Before insect host death, fungal infection can result in reduction of feeding and reproduction potential [16, 17].

Therefore, the complementary action of EPF on tsetse mortality on the reduction of blood feeding and reproduction potential, and possible effects on the development of the trypanosome in the vector could play an important role in the epidemiology of the disease in humans and cattle by limiting its spread. This study aimed to investigate the interactions between the entomopathogenic fungus *Metarhizium anisopliae,* the parasite *Trypanosoma congolense*, Broden 1904, and the vector tsetse fly *Glossina fuscipes fuscipes.* Specifically, we wanted to investigate the effect of fungal infection by *M. anisopliae* on the reproduction of *T. congolense* in *G. f. fuscipes* and on the vectorial capacity to acquire and transmit the disease causing organism.

## Methods

### Insects and mice

Tsetse flies, *Glossina fuscipes fuscipes,* used in this study were collected from Ungwe Valley, Mbita, Kenya. The flies were reared in vivo on rabbit blood at the Animal Rearing and Containment Unit (ARCU) at the International Centre of Insect Physiology and Ecology (ICIPE), under controlled conditions of 25 ± 2 °C, at 70% relative humidity (RH) under a photoperiod of 12 h light (L):12 h dark (D) (12:12 h L:D).

Swiss albino mice (*Mus musculus*) were also reared at the Animal Rearing and Containment Unit under controlled conditions.

### Parasites

*Trypanosoma congolense* (strain ICIPE 002) population used in this study were obtained from ICIPE’s Molecular Biology and Bioinformatics Unit where it was stored in liquid nitrogen at − 196 °C. The trypanosome strain was isolated from flies collected in Shimba Hills, Kenya, and propagated in Swiss mice that were immuno-suppressed for 24 h using cyclophosphamide (Sigma Aldrich, GmbH, Germany) to increase the rate of infection.

### Fungal cultures

Two strains of *M. anisopliae* sensu *lato* (Khamis et al., unpublished data) used in the study were ICIPE 30 wild type (WT 30) and ICIPE 30 transformed to express green fluorescent protein (GFP) (named GZP-1). The latter strain was transformed and provided to us by R. St. Leger (University of Maryland, USA). The cultures were maintained on Sabouraud Dextrose Agar (SDA) at 25 ± 1 °C and 12:12 h L:D photoperiod. Conidia were collected by scrapping two-week old sporulating cultures under aseptic conditions.

### Contamination of flies with entomopathogenic fungi

**Fig. 1:**
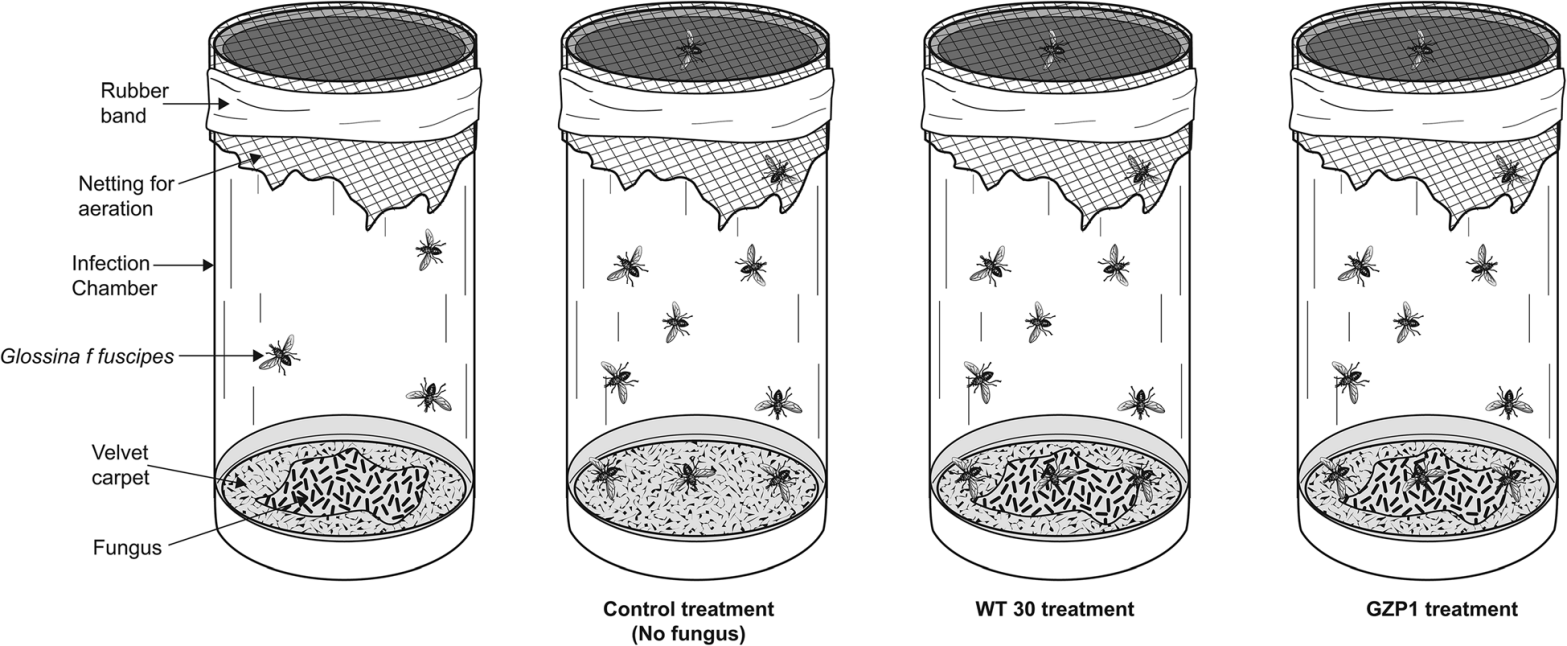
Diagrammatic representation illustrating the technique (infection chamber) used in infecting the tsetse flies with fungus in the assays

Experimental-insects were infected using velvet carpet fabric layered with conidia that covered the inside of a cylindrical plastic tube (infection chamber) (95 × 48 mm) and had white nylon netting over one end (Fig. 1), as described by Dimbi et al. [18]. This technique used was initially developed for fruit flies, and found suitable for tsetse flies. Dry conidia (0.1 g) were spread evenly onto the velvet material. Ten flies were enclosed within the infection chamber and exposed to the fungus for 12 h after which they were transferred and placed in clean cylindrical plastic tubes (95 × 48 mm) and maintained at 25 ± 2 °C at 55% RH, in an incubator in the lab under a 12:12 h L:D photoperiod.

### *Trypanosoma* infection of fly

Parasite infection of flies was accomplished by using a silicone membrane feeding technique with sterile pig blood as described by Moloo [19]. Before inoculation with trypanosomes, the blood was tested and cleared of any bacteria present using standard procedures established at the ARCU prior to being amended with 10 mM final concentration of L-glutathione (Sigma, GmbH, Aldrich, Germany) and 4 × 10^6^ trypanosomes per ml of blood. The L-glutathione increases flies’ susceptibility to trypanosome infection [20].

### Effects of fungal infection on parasite reproduction

Forty-eight (48) h-old teneral adult tsetse flies were fed with trypanosome-infected pig blood for 2 h, then exposed to conidia in the infection chamber (Fig. 1) for 12 h, on the following day. In the control treatment, flies were exposed to velvet carpet fabric devoid of conidia. All the flies were dissected on day 2, 3, 5 and 7 following treatment for the presence of the parasite in the midgut. The alimentary tracts were dissected on glass slides containing a drop of phosphate buffered saline and viewed as wet mounts under a phase contrast microscope at 100× magnification for detecting trypanosomes. The midgut and proboscis were separated, dissected and then either examined via bright field microscopy or assayed through polymerase chain reactions (PCR) (see following sections). Treatments were randomized and consisted of 10 flies per replicate and the experiment was repeated three times.

### Effects of fungal infection on parasite acquisition by tsetse flies

Two-day-old fungus-inoculated flies (either males or females) were offered trypanosome-contaminated blood meal for 10 min (via silicone membrane as described above) every two-day. In the control treatment, flies were not exposed to fungal treatments (were exposed to velvet devoid of conidia). Flies were then dissected as described by Lloyd et al. [21] to determine parasite acquisition. Treatments were also randomized and consisted of 10 flies per replicate and the experiment was repeated three times.

### Effects of fungal infection on parasite transmission by tsetse flies

Two-day old tsetse teneral flies (either males or females) were fed on trypanosome-infected blood for 2 h and thereafter maintained under controlled temperature and relative humidity (25 ± 2 °C, 70% RH) with 10-min regular feeding for 11 days on clean blood to allow for parasite development. On day 12, the test-flies (in cohorts of 10) were exposed to fungal treatments for 12 h (as described above) and later fed on trypanosome-free mice daily for 10 min for a period of 7 days. The mice were immobilized in a small cage and the flies in a cylindrical tube with netting over one end placed underneath the mice to enable flies to feed. Parasitemia (the demonstrable presence of parasites) in mice was monitored daily for 7 days through a tail prick to obtain a drop of blood for examination under the microscope (100×). Quantification of trypanosome was achieved by matching what was observed in the microscopic field of a wet blood film with the charts and by counting trypanosomes in 5, 10 or 20 microscopic fields as described by Herbert and Lumsden [22]. Treatments were randomized and consisted of 10 flies per replicate and the experiment was repeated three times. The Swiss mice used were immuno-suppressed for 24 h using cyclophosphamide (Sigma Aldrich, GmbH, Germany) prior to the assay.

### Molecular quantification of the trypanosomes

Traditionally, trypanosomes have been detected in insects by direct microscopic examination of gut contents or dissection and analysis of the entire fly. However, this microscopic observation has proved to be laborious, requiring extensive time for extrusion of the abdomen, mounting and scanning per insect. In addition, microscopic detection of procyclic trypomastigotes is challenging; therefore, PCR-based detection from ingested blood samples provided a highly sensitive method for detecting low parasite numbers. Through quantitative PCR the infection status and titers of trypanosomes in individual insects can be estimated simultaneously [23].

Genomic DNA was extracted from the proboscis, proventriculus and midgut from each of the infected and control flies (a total of 30 samples) using the Isolate II Genomic DNA Kit (Bioline, UK) following the manufacturer’s protocol. The quality of extracted DNA was checked using Nanodrop spectrophotometer (ND 2000c Thermo Scientific, Wilmington, USA). Samples were stored at − 20 °C until used for downstream processes. The presence of trypanosome DNA was probed through amplification via universal (ITS 1 CF/BR) and species-specific (TCS_F/R) primers. The TCS_F/R markers were used on all samples because they are more stringent when trypanosome titers are low. The intergenic transcribed spacer region (ITS) was targeted in a final reaction volume of 20 μL consisting of 5X My *Taq* Reaction Buffer (5 mM dNTPs, 15 mM MgCl_2_, stabilizers and enhancers) (Bioline, London, UK), 0.5 μmole μL^− 1^ of each primer (ITS 1 CF 5’ CCGGAAGTTCACCGATATTG 3’and ITS 1 BR 5’ TTGCTGCGTTCTTCAACGAA 3′) [24], 1.25 Units My *Taq* DNA polymerase (Bioline, London, UK) and 15 ng of DNA template. This reaction was set up in the Arktik thermal cycler (Thermo Fisher Scientific, Waltham, Massachusetts, USA) using the following cycling conditions: initial denaturation for 2 min at 95 °C, followed by 40 cycles of 30 s at 95 °C, 30 s at annealing temperature of 53.7 °C and 30 s at 72 °C, then a final elongation step of 10 min at 72 °C. A specific satellite DNA monomer of *T. congolense* was targeted using species-specific TCS_F (5’ CGAGAACGGGCACTTTGCGA 3′) and TCS_R (5’ GGACAAACAAATCCCGCACA 3′) [25] primers. The PCR conditions were the same except that the annealing was done at 60 °C for 30 s. PCR products resolved in a 1.5% agarose gel were stained and documented under ultraviolet (U.V) trans-illuminator using the KETA GL imaging system (Wealtec Corp, Meadowvale Way Sparks, Nevada, USA).

The parasite titers were estimated using the *T. congolense* Savannah specific primers via qPCR in a final reaction volume of 12.5 μL consisting of 2X Maxima SYBR Green/ROX qPCR Master Mix (Thermo Fischer Scientific, Waltham, Massachusetts, USA), 0.5 μmole μL^− 1^ of each primer (TCS), and 15 ng of DNA template. The reactions were carried out in a real-time qPCR machine (Strategene MX3005P, Agilent Technologies, CA, USA) per the manufacturer’s instructions. The thermo-cycling conditions involved an initial step of 95 °C for 10 min, 40 cycles of 95 °C for 15 s, 60 °C for 30 s and 72 °C for 30 s, followed by one cycle of 95 °C for 30 s, 60 °C for 1 min and 95 °C for 30 s. *Glossina* glyceraldehyde-3-phosphate dehydrogenase GAPDH (GenBank: DQ016434.1) gene was used as internal control in the qPCR assays.

### Data analysis

Student’s *t*-test at CI = 0.95 was used to compare significant differences between the two fungal treatments (WT 30 and GZP-1). The presence of *T. congolense* in the infected-flies’ midgut was estimated by comparing wet microscopic fields as described by Herbert and Lumsden [22]. One-way analysis of variance using *F*-distribution (*F*-test) by Fisher was used to analyze data on pathogen multiplication, ability of flies to acquire *T. congolense* and mortality between males and females (*P* < 0.05). Significant differences between the male and female flies response to the treatments with regards to their ability to acquire trypanosome parasite *T. congolense* were assessed using Chi-square test at *P* ≤ 0.05. Proportion of parasite transmission to mice by the flies was categorized as either being present or absent, as this could not be quantified. The Kolmogorov-Smirnov test (*P* < 0.05) was used to analyze the raw crossing threshold (Ct) values which were then used to quantify expression of *T. congolense* satellite monomer DNA using the comparative Ct (2^-∆∆Ct^) method [26]. Mann – Whitney *U*-test was applied using the GraphPad Prism version 7.00 for Windows (GraphPad Software, La Jolla, CA) (www.graphpad.com), to determine probabilities of *P* < 0.05 as significant, to calculate the statistical significance of the Ct differences between the treatments.

## Results

### Effects of fungal exposure on parasite multiplication

**Fig. 2:**
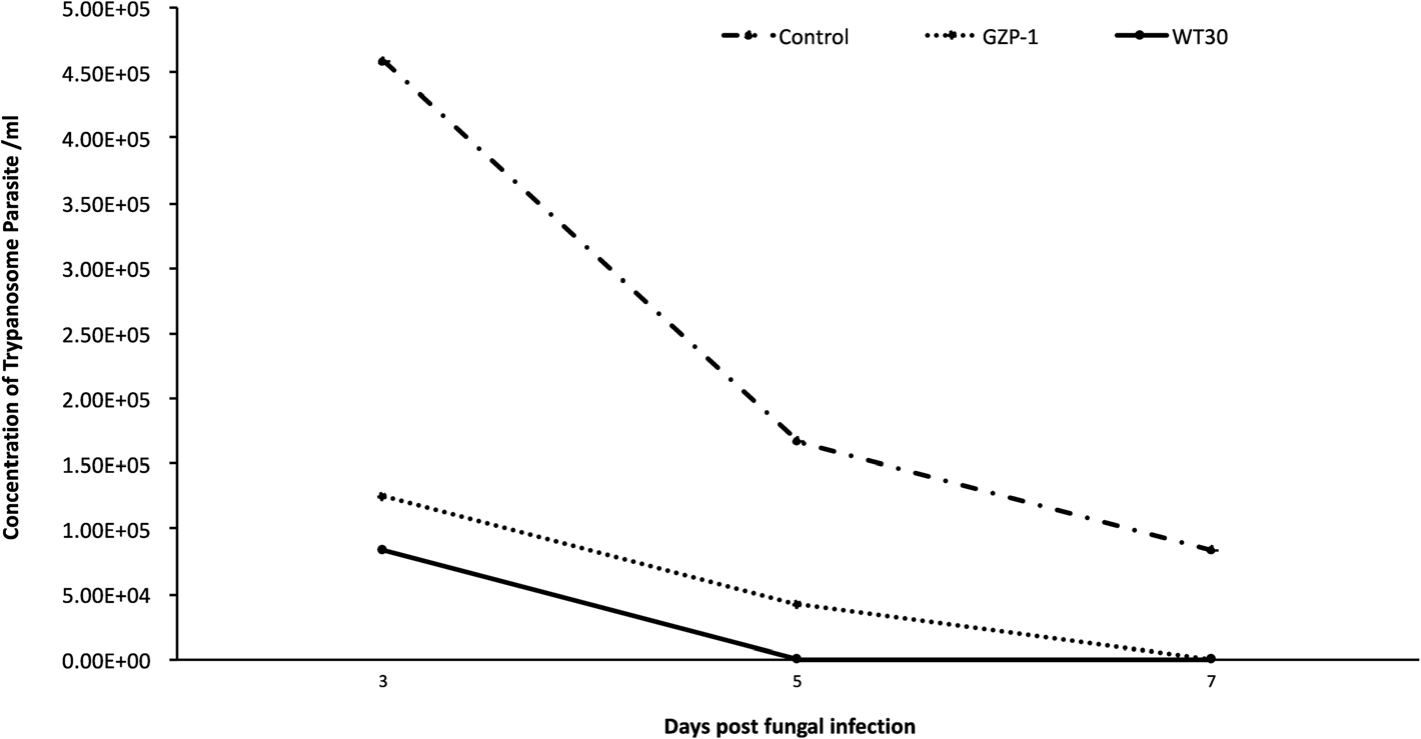
Effects of fungal treatment on the *Trypanosoma congolense* parasite concentration in *Glossina fuscipes fuscipes* midgut, three, five and seven-day post-exposure

The concentration of the trypanosome parasites in infested blood was 1.1 ± 0.1 × 10^8^ ml^− 1^ in all the treatments. There were significant differences in the *T. congolense* parasite load in *G. f. fuscipes* at day 3 (*F* = 15.32, df = 2, 15; *P* < 0.0001), day 5 (*F* = 34.33, df = 2, 15; *P* < 0.0001) and day 7 (*F* = 9.87, df = 2, 15; *P* = 0.007) post-fungal exposure (Fig. 2). At day 3 post-fungal exposure, parasite concentration was high in the control (4.6 ± 0.1 × 10^5^ ml^− 1^) while it was significantly reduced in GZP-1 (1.3 ± 0.7 × 10^5^ ml^− 1^) and WT 30 (8.3 ± 0.6 × 10^4^ ml^− 1^) (Fig. 2). At day 5 post-fungal exposure, the concentration of *T. congolense* parasite was 1.7 ± 0.8 × 10^5^ ml^− 1^ in the control while it was drastically reduced in GZP-1 (4.2 ± 0.5 × 10^4^ ml^− 1^). No parasites were observed in WT 30 treated flies 5 day post-fungal exposure (Fig. 2).

Only living flies were examined to assess the parasite load. The reduction of the parasite concentration in the flies was more than 13 fold over the control; however, *T. congolense* parasite mortalities recorded in the fungal treatments were not significantly different (*F* = 0.45, df = 2, 9; *P* = 0.653) compared to the control. Similarly, no significant difference (*F* = 1.43, df = 2, 9; *P* = 0.288) was observed between the dead host flies across treatments. Additionally, the lethal time mortality (LT_50_) values of the host flies were recorded and varied between 3.5 and 7 days in the fungal treatments.

**Fig. 3:**
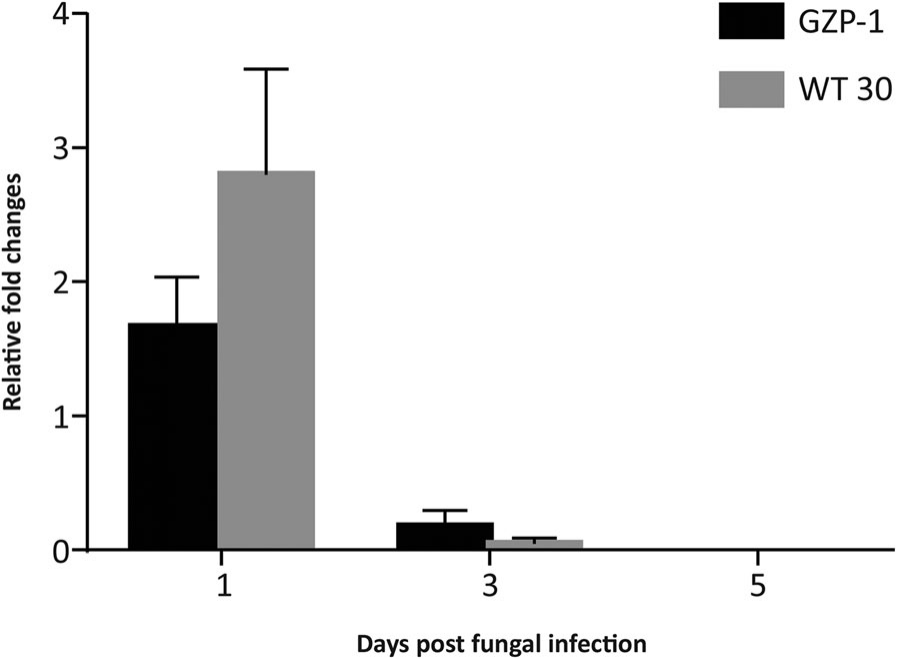
Relative *Trypanosoma congolense* satellite DNA monomer gene expression in the flies inoculated with GZP-1 and WT 30

Similar trends of the *T. congolense* parasite load as observed in Fig. 2 were confirmed via quantitative PCR. Quantitative PCR showed no significant difference between GZP-1 and WT 30 treatments in parasite titers over time (Fig. 3). However, as the days progressed, there was a significant (*F* = 24.42, df = 2, 30, *P* < 0.0001) drop in *T. congolense* titers in the flies infected with fungi as shown by the relative fold changes (target gene expression) of the parasite satellite monomer DNA (Fig. 3).

### Effects of fungal exposure on parasite acquisition by flies

**Fig. 4:**
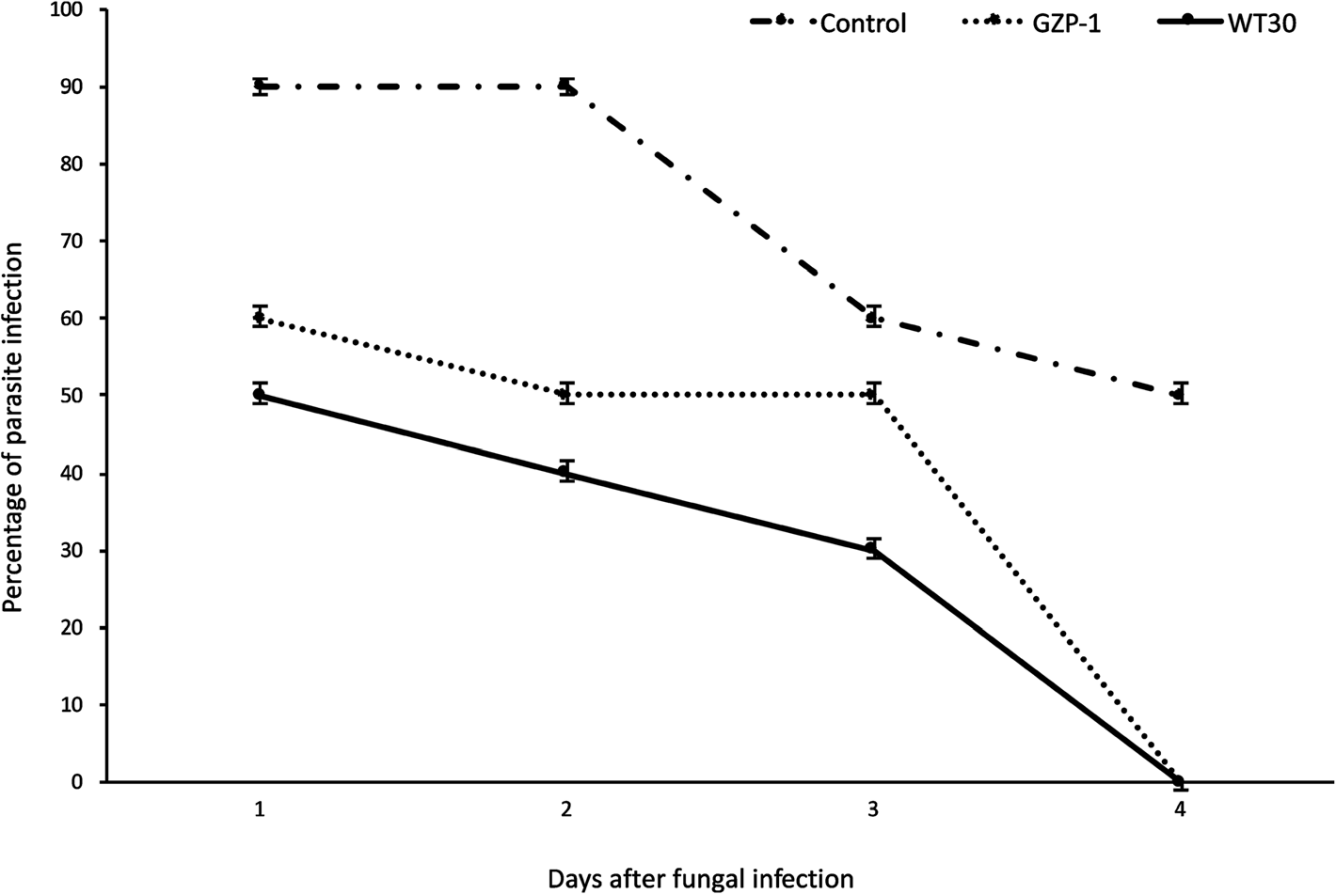
Percentage of *Trypanosoma congolense* acquired by *Glossina fuscipes fuscipes* flies following fungal infection by *Metarhizium anisopliae* WT and *M. anisopliae* GZP-1

When flies were offered trypanosome*-*infected blood, they acquired *T. congolense* in both the control and fungal treatments, from day 1 to day 3 (Fig. 4). However, the percentage of flies that acquired parasites 2 days post-exposure was significantly higher (*F* = 9.26, df = 2, 27; *P* = 0.0054) in the control than those exposed to the fungal treatments, whereby 90% of flies acquired parasites in the control, compared to only 50 and 60% in WT 30 and GZP-1 treatments, respectively (Fig. 4). In addition, flies in the control treatment continued to acquire *T. congolense* on day 4 while no parasite acquisition was observed in either of the fungal treatments (Fig. 4).

There were no significant differences between percent male and female flies acquiring parasites in the GZP-1 treatment (χ^2^ ranged between 0.85–3.24; and *P* values were ranged between 0.07186–0.1282) and control treatment (χ^2^ ranged between 0.00–1.47; and *P* values were ranged between 0.2249–1.00) (Table 1). However, the acquisition of parasites for both sexes combined in the control was at least 30% higher compared to the GZP-1 fungus-treated flies 2–4 days post-exposure.

**Table 1:** Effects of infection by *Metarhizium anisopliae* GZP-1 on male and female *Glossina fuscipes fuscipes* ability to acquire *Trypanosoma congolense* after 4 days post-exposure. Only *M. anisopliae* GZP-1 was used for this study

Treatments	Sex ratio	Day 1	Day 2	Day 3	Day 4
GZP-1	% Male	100a	50a	30a	20a
	% Female	90a	60a	40a	30a
χ^2^		0.85	1.47	2.31	3.24
P value		0.3558	0.2249	0.1282	0.07186
Control	% Male	100a	90a	70a	50a
	% Female	100a	90a	80a	60a
χ^2^		0.00	0.00	1.08	1.47
*P* value		1.00	1.00	0.2987	0.2249

Within columns and for each treatment, means followed by the same lower case letters are not significantly different at P < 0.05 (Chi-square test)

### Effects of fungal treatments on parasite transmission by flies

When *T. congolense-*infected flies were exposed to fungal treatments with *M. anisopliae* WT 30 and GZP-1 strains, they were unable to transmit the parasite to naïve mice (mice that hadn’t been exposed to any trypanosome parasites before), while control *Trypanosoma*-infected flies continued to transmit the parasite to naïve mice (Table 2). The results showed that *M. anisopliae* WT 30 and GZP-1 strains blocked the transmission of *T. congolense* parasite in mice from day 1 to 7 days post-fungal treatment.

**Table 2:** Effect of *Metarhizium anisopliae* infection of *Glossina fuscipes* flies on transmission of *Trypanosoma congolense* in mice

	Presence/Absence of *T. congolense* in mice			
	Days after exposure to fungal infection of flies with trypomastigotes in their proboscis (11-day-old parasite-infected flies)			
Treatment	Day 1	Day 3	Day 5	Day 7
Control	+	+	+	+
*M. anisopliae* 30 (WT 30)	–	–	–	–
*M. anisopliae* 30 (GZP-1)	–	–	–	–

NB: +, Presence of *T. congolense*; −, Absence of *T. congolense*

## Discussion

The present study has demonstrated that fungal infection by *M. anisopliae* affects the reproduction of *T. congolense* in *G. f. fuscipes* and also the vectorial capacity of these flies to transmit the trypanosome parasite. The parasitemia titers observed through microscopic observation were confirmed by qPCR, whereby the relative *T. congolense* satellite DNA monomer gene expression in the flies inoculated with GZP-1 and WT 30 showed a decreasing trend in trypanosome numbers over time. This finding was in tandem with the hypothesis that when flies infected with trypanosomes are exposed to fungal exposure, the trypanosome titers are significantly reduced. Parasitemia load decreased in all the treatments including control at day 3 post-treatment; however, no parasitemia was observed after day 4 post-treatment in either fungal treatment which may correlate with the period of time required for the fungal infection to kill the flies. Lethal time mortality (LT_50_) values of between 4.0 and 7.5 days (depending on the concentration) has been reported on another species of tsetse fly, *Glossina morsitans centralis,* with the same fungal isolate (*M. anisopliae*) [27]. The midgut is considered as a preferred site for the development of *T. congolense* before migrating to the proboscis [28]. In mosquitoes, Fang et al. [29] observed low prevalence and density of sporozoites following infection with wild-type strain of *Metarhizium anisopliae* 11 days after a *Plasmodium*-infected blood meal. In this study, *G. fuscipes fuscipes* flies previously infected with *T. congolense* for 11 days (presumably the necessary time for the parasite to migrate into the proboscis and become infective) and exposed to the fungal treatment *M. anisopliae* WT 30 and GZP-1 strains on the 12th day (i.e. 1 day post-fungal exposure but 11 days after flies were fed on trypanosome-infected blood), were unable to transmit trypanosome parasites to mice following fungal exposure while flies in the control continued to successfully transmit the parasite during the whole experimental period.

Transmission of the parasite to the host is achieved through the bites of a *Trypanosoma-*infected tsetse fly while taking a blood meal. In mosquitoes for instance, reduction in blood meal intake following infection by entomopathogenic fungus *M. anisopliae* has been associated with reduction in transmission potential of the parasite [29–31]. In contrast, we found that over 65% of fungus-infected *G. fuscipes fuscipes* flies were still able to feed after 3 days’ post-fungal infection; therefore, behavioral changes alone cannot explain these results. We hypothesize that other interactions between the fungus and the parasite within the host may account for these observational differences. For instance, entomopathogenic fungi must overcome and or avoid host immune defenses during infection process by production of metabolites in the insect haemocoel. Some of the metabolites such as destruxins have been shown to have antiviral properties in insect and human cell lines [32]. The effect of fungal metabolites on trypanosome parasites has to be investigated.

No significant differences were observed in tsetse flies’ male and female ability to acquire *T. congolense* in the fungal treatments, which indicates that both sexes can acquire the parasite equally after infection. In the present study, we were unable to demonstrate coexistence of *M. anisopliae* propagules and *T. congolense* in infected flies midgut and proboscis as was shown to be the case for *M. anisopliae* and *Plasmodium falciparum* in the hemolymph of infected mosquitoes [29].

## Conclusions

Our study has shown that both the reproduction of the parasite, *T. congolense* within its insect host (*G. fuscipes fuscipes*) and the fly’s vectorial capacity to acquire or transmit the parasite was negatively affected by the insect pathogenic fungus, *M. anisopliae*. Furthermore, more *s*tudies are needed to elucidate the mechanisms of insect-fungus interaction observed in this study. Understanding the role of metabolites, i.e. destruxins, swainsinone, and cytochalasin C may be important in determining the virulence and/or specificity against insects, especially as it relates to other *Glossina* species.

## Abbreviations

AAT: Animal African trypanosome
EPF: Entomopathogenic fungi
HAT: Human African trypanosome
ICIPE: International Centre of Insect Physiology and Ecology
RH: Relative humidity
WT: Wild type
